# Extended Human-to-Human Transmission during a Monkeypox Outbreak in the Democratic Republic of the Congo

**DOI:** 10.3201/eid2206.150579

**Published:** 2016-06

**Authors:** Leisha Diane Nolen, Lynda Osadebe, Jacques Katomba, Jacques Likofata, Daniel Mukadi, Benjamin Monroe, Jeffrey Doty, Christine Marie Hughes, Joelle Kabamba, Jean Malekani, Pierre Lokwa Bomponda, Jules Inonga Lokota, Marcel Pie Balilo, Toutou Likafi, Robert Shongo Lushima, Benoit Kebela Ilunga, Frida Nkawa, Elisabeth Pukuta, Stomy Karhemere, Jean-Jacques Muyembe Tamfum, Beatrice Nguete, Emile Okitolonda Wemakoy, Andrea M. McCollum, Mary G. Reynolds

**Affiliations:** Centers for Disease Control and Prevention, Atlanta, Georgia, USA (L.D. Nolen, L. Osadebe, B. Monroe, J. Doty, C.M. Hughes, A.M. McCollum, M.G. Reynolds);; Minstere de la Santé, Kinshasa, Democratic Republic of the Congo (J. Katomba, J. Likofata, R. Shongo Lushima, B. Kebela Ilunga);; US Centers for Disease Control and Prevention, Kinshasa (J. Katomba, J. Likofata, J. Kabamba);; National Institute for Biomedical Research, Kinshasa (D. Mukadi, F. Nkawa, E. Pukuta, S. Karhemere, J.-J. Muyembe Tamfum);; University of Kinshasa, Kinshasa (J. Malekani,);; Kinshasa School of Public Health, Kinshasa (M.P. Balilo, T. Likafi, B. Nguete, E. Okitolonda Wemakoy);; Minstere de la Santé Tshuapa Health District, Bokungu, Democratic Republic of the Congo (P.L. Bomponda, J.I. Lokota)

**Keywords:** monkeypox, monkeypox virus, viruses, transmission, incubation, animal diseases, Africa, orthopoxvirus, zoonosis, Democratic Republic of the Congo

## Abstract

A 600-fold increase in monkeypox cases occurred in the Bokungu Health Zone of the Democratic Republic of the Congo during the second half of 2013; this increase prompted an outbreak investigation. A total of 104 possible cases were reported from this health zone; among 60 suspected cases that were tested, 50 (48.1%) cases were confirmed by laboratory testing, and 10 (9.6%) tested negative for monkeypox virus (MPXV) infection. The household attack rate (i.e., rate of persons living with an infected person that develop symptoms of MPXV infection) was 50%. Nine families showed >1 transmission event, and >6 transmission events occurred within this health zone. Mean incubation period was 8 days (range 4–14 days). The high attack rate and transmission observed in this study reinforce the importance of surveillance and rapid identification of monkeypox cases. Community education and training are needed to prevent transmission of MPXV infection during outbreaks.

## Abstract 2

During the outbreak, 50% of household members living with an infected person developed symptom of monkeypox infection.

Monkeypox virus (MPXV), which belongs to the genus *Orthopoxvirus*, is zoonotic and endemic to western and central Africa. MPXV is a close relative of the variola virus, and monkeypox illness resembles a smallpox-like infection but is less severe than smallpox. Most patients initially develop a fever, followed by rash a few days later. Lymphadenopathy is a common sign and occurs just before or concomitant with the rash (*1**,**2*). Up to 11% of unvaccinated affected persons die (*3*). No targeted medications are licensed to treat this infection. Although smallpox vaccination can provide some protection against infection, this vaccination is not used in MPXV-endemic areas because of cost considerations and safety concerns about using a vaccine that contains live vaccinia virus.

MPXV transmission among close contacts within households is well documented; previous reports have shown up to 6 intrafamily transmission events (*4*). Transmission is thought to occur by means of salivary or respiratory droplets or contact with lesion exudate (*5**,**6*); however, evidence suggests that infection can occur by direct inoculation (*7*). Previous household attack rates (i.e., rates of persons living with an infected person and developing symptoms of MPXV infection) of 3%–11% have been reported (*6**,**8*). Although some reports show a high incidence of households with single isolated cases (*8*), other reports have documented frequent transmission events within households (*6**,**9*). Attack rates have been found to be much higher among persons living in households with an MPXV patient and among persons with no evidence of prior smallpox vaccination (*6**,**8*).

Monkeypox is a reportable disease in the Democratic Republic of the Congo (DRC), and cases are reported from 26 health districts (containing 512 health zones). During 2013, a substantial increase in the number of suspected human monkeypox cases was noted in the Bokungu Health Zone within Tshuapa District of DRC’s Equateur province (Figure 1). In December 2013, we conducted an investigation of monkeypox for this health zone and focused on cases reported during July 1–December 8, 2013.

**Figure 1:**
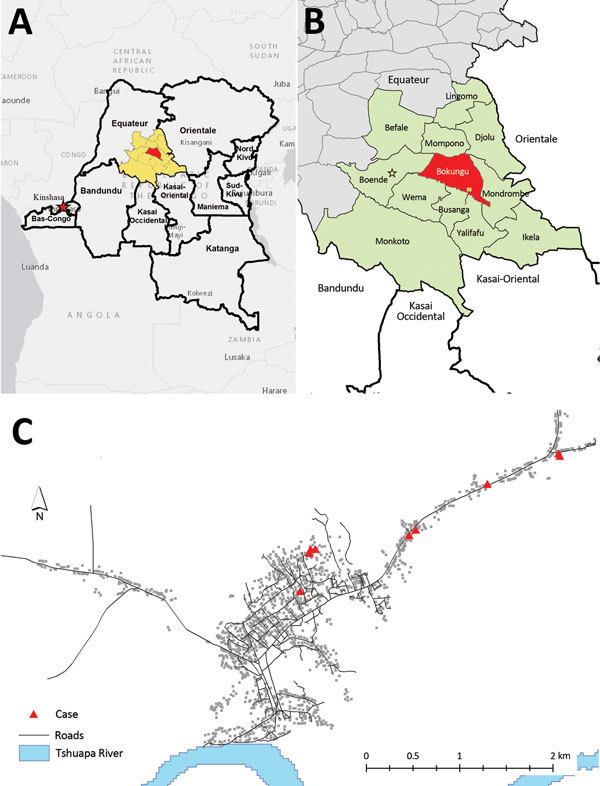
Region affected by monkeypox illness. A) The Democratic Republic of the Congo is outlined; Tshuapa District is highlighted in yellow and Bokungu Health Zone in red. B) Health zones within Tshuapa District; Bokungu Health Zone is highlighted in red. The village with the largest cluster of cases is indicated by a yellow square. C) Distribution of cases (shown by red triangles) in the village with the most cases during this outbreak.

## Methods

DRC has a regional surveillance system that collects reports of all suspected monkeypox cases. When possible, cases are investigated, a monkeypox-specific case report form is completed, and replicate diagnostic specimens (derived from lesions) are collected. During this investigation, additional retrospective cases within affected villages and households were identified on the basis of physical symptoms that were reported by patients and family members but that had not previously reported to the surveillance system.

### Case Definitions

Cases in our investigation must have occurred during July 1–December 8, 2013. We used the following case definitions as part of the enhanced surveillance system in Tshuapa District. A confirmed case occurred in a person with a history of high fever, a vesicular-pustular rash, and >1 of the following 3 characteristics: 1) rash on the palms and soles, 2) lymphadenopathy, 3) fever preceding rash. In addition, this person has a PCR-tested diagnostic specimen that yielded a positive result for *Orthopoxvirus* or had MPXV DNA signatures. A probable case occurred in a person with a history of high fever, a vesicular-pustular rash, and >1 of the following 3 characteristics: 1) rash on the palms and soles, 2) lymphadenopathy, 3) fever preceding rash. In addition, this person has a history of contact with a person or animal with confirmed monkeypox during the 14 days before illness onset. A suspected case occurred in a person with a history of high fever, a vesicular-pustular rash, and >1 of the following three characteristics: 1) rash on the palms and soles, 2) lymphadenopathy, 3) fever preceding rash.

Clinical symptoms were documented by a trained healthcare provider or investigator who used a standardized case-reporting form. Smallpox vaccination status was documented on the basis of patient recall and presence of a vaccination scar on the left upper arm. Analysis of vaccination status was performed with χ^2^ test by using STATA 14.0 (https://www.stata.com/). Age was considered dichotomous because vaccination was available only to those >33 years of age, as a result of the discontinuation of smallpox vaccination in DRC (and other countries) 33 years earlier (1980) after the announcement of smallpox eradication.

Diagnostic specimens (crusts, vesicular fluid, or ocular fluid) were collected and shipped to the Institut National de Recherche Biomedicale in Kinshasa, DRC, for analysis. DNA was extracted from each specimen, and an *Orthopoxvirus*-specific real-time PCR assay (*10*) was performed for diagnostic confirmation. If no *Orthopoxvirus* DNA was amplified, then a second real-time PCR assay was performed for varicella zoster virus (VZV)–specific DNA (US Army Medical Research Institute of Infectious Diseases, unpub. data).

### Symptom Intervals

Incubation period was defined as the number of days between contact with a symptomatic monkeypox patient and development of rash. Rash was chosen as the benchmark of infection for estimating incubation periods because families were better able to recall the day of rash onset than to recall the day of fever onset. To obtain the best estimate of the MPXV incubation period, we identified patients who reported clear dates of exposure and rash onset in our investigation and in the published literature (*4**,**9**,**11*). We determined a mathematical distribution of incubation times and calculated the mean, median, and range for the central 75% of the cases (that is, we excluded data for patients at either end of the distribution). 

We conducted a larger analysis that combined the data from those persons with clearly identified dates of exposure with data containing household transmission intervals. The household data was calculated by determining the time between onset of rash for the first and second cases in a household. Cases were eliminated from this analysis if the first 2 cases in a household were separated by <3 days because we assumed that these case-patients were infected by the same source. We determined a mathematical distribution for the incubation times of this larger group. A secondary analysis of the dataset containing only persons with clearly defined dates of exposure and the dataset which included household transmission was performed by using an alternative formula that was developed to model serial case intervals for respiratory infections (*11*).

### Transmission Chains

We estimated transmission chains (i.e., a series of persons who sequentially pass the infection to the next person) within families and villages on the basis of the calculated incubation periods for household transmission. Cases were considered independent when the interval between the onset of rash for a case-patient in the household or village was >8 days from the onset of symptoms for the previous case-patient. This value was chosen because we assumed that the first case occurred after the shortest possible incubation period (5 days) and that the last possible case occurred after the longest possible incubation period (13 days). Any cases occurring after this window of 5–13 days are considered to result from an independent infection either inside or outside the household. Coordinates for case households were recorded with handheld global positioning system units (eTrex 10; Garmin, Olathe, KS, USA) and compiled with the locations of residential structures digitized from satellite images (DigitalGlobe, Westminster, CO, USA). Household counts were aggregated into a 50-square-meter grid covering the entire populated area. Tests for spatial autocorrelation were performed by using the Global Moran’s I tool in ArcGIS 10.2 (ESRI, Redlands, CA, USA) at distances of 50–1,000 meters in 50-meter increments.

## Results

### Monkeypox in Bokungu Health Zone

During 2013, a total of 104 suspected case-patients with human MPXV infection and 10 deaths (9.6%) were reported from the Bokungu Health Zone to the national surveillance system, with October showing a dramatic increase in number of cases (Table 1). Of the 104 suspected case-patients, 60 (57.7%) had active lesions, and specimens were collected from these persons for testing. Of tested specimens, 50 (83.3%) were confirmed MPXV infections. Because MPXV infection and VZV infection have clinical similarities, testing for VZV was also performed. Five (8.3%) of the 60 patients had specimens that tested positive for VZV, and specimens for 5 (8.3%) failed to yield a positive result for either virus. 

**Table 1:** Reported monkeypox cases and deaths by month, Bokungu Health Zone, 2013*

Cases and deaths	Jan	Feb	Mar	Apr	May	Jun	Jul	Aug	Sep	Oct	Nov	Dec	Total
Cases, no.	3	0	2	3	0	10	6	0	1	61	2	16	104
Deaths, no.	0	0	0	0	0	1	0	0	0	8	0	1	10
*All cases, not yet characterized as confirmed, probable, or suspected.													

**Figure 2:**
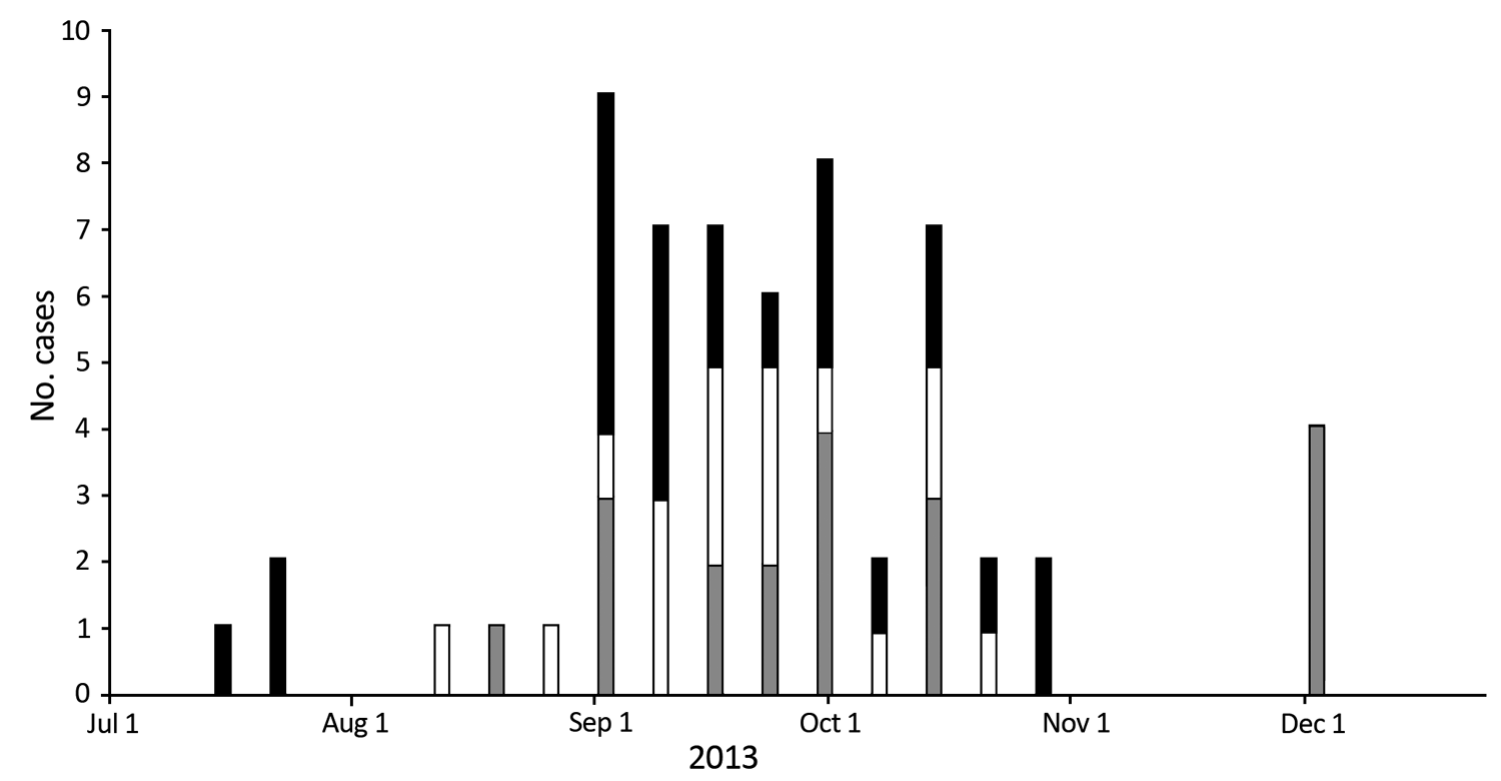
Epicurve of cases included in investigation and monkeypox cases during investigation period (July 1–December 8, 2013). Black represents suspected cases, white represents probable cases, and gray represents confirmed cases.

During the focused investigation period (July–December 2013), we identified and interviewed 63 case-patients in 16 households (Table 2). Of these case-patients, 26 had previously been identified, investigated, and reported by local health authorities; our investigation identified an additional 37 case-patients, including 4 with acute illness. Of the total 63 case-patients, 20 were confirmed, 19 were probable, and 24 were suspected cases. Median age of case-patients was 10 years (range 4 months–68 years); 17.7% were <5 years of age (Table 2). Of the 63 case-patients, 36 (57.1%) were male. Most cases occurred within a 74-day period between the first week of September and the last week of October (Figure 2). All 63 cases included in the 6-month investigation occurred within a 144-day window.

**Table 2:** Characteristics of patients with monkeypox infections, Bokungu Health Zone, July–December 2013

Characteristic	Total cases, N = 63	Confirmed cases, n = 20	Probable cases, n = 19	Suspected cases, n = 24	Unaffected household members,* n = 53
Median age, y (mean)	10 (15.5)	14 (20.4)	7 (6.7)	10 (16.4)	20 (23)
Age range	4 mo–68 y	8 mo–68 y	4 mo–21 y	6 mo–65 y	2 mo–72 y
Male sex, no. (%)†	36 (57)	12 (60)	9/18 (50)	15/22 (68)	19/50 (38)
Vaccinated, no. (%)†	9/59 (15)	5/18 (28)	0/18 (0)	4/23 (17)	14/53 (26)
*Persons in households without symptoms and not tested. †Denominators indicate no. patients with data available in that category.					

In the 16 investigated households, 9 (15%) of affected household members had evidence of a prior smallpox vaccination, compared with 30% of unaffected household members; χ^2^ analysis showed that that this difference was not significant (p>0.05). However, vaccination status and age >33 years were nearly perfectly correlated (p< 0.001).

The median number of persons affected within each household was 3 (mean 3.9; range 1–8). The median attack rate within households was 50%; mean was 52.1% (range 50%–100%). For 1 of the 16 families investigated, all 6 household members were affected. For all households, the median interval between the time that rash developed in the first person in the household to time that rash developed in the last person was 10 days (range 2–41 days).

### Incubation Period

**Figure 3:**
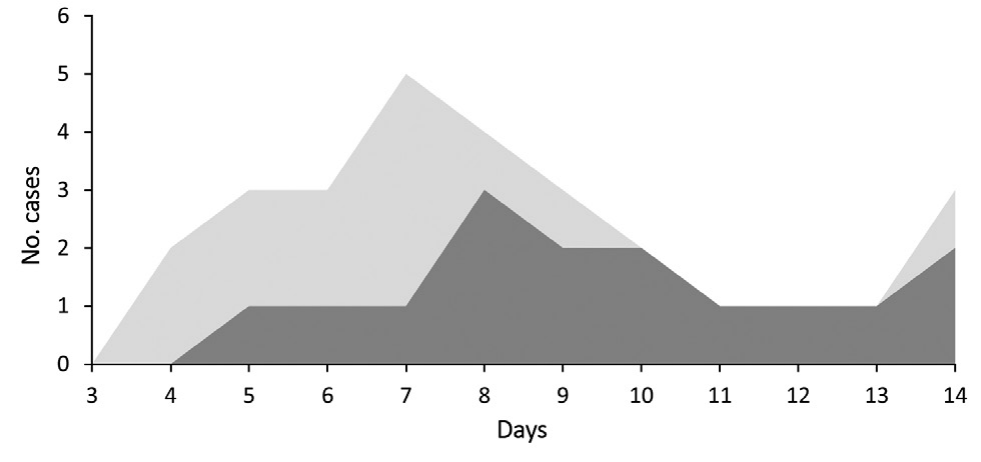
Distribution of incubation periods from 2 separate analyses. Dark gray shows the distribution of incubation periods on the basis of case-patients with well-defined dates of exposure identified in our investigation and in the published literature (n = 16). Light gray shows the distribution of incubation periods from the literature and incubation periods calculated by using the first 2 case-patients in each family (n = 28).

Four case-patients were able to identify a specific date of monkeypox exposure and rash onset. These persons reported that rash developed 5–8 days after contact with an earlier case-patient. A PubMed (http://www.ncbi.nlm.nih.gov/pubmed) search identified 12 additional persons who had confirmed or probable infection and well-defined incubations periods; these case-patients had an incubation period of 9–14 days (*4**,**9**,**11*). When the 4 case-patients in our investigation and the 12 historical case-patients were considered together, mean incubation period for all was 9.6 days and median was 9 days. The central 75% of these case-patients had an incubation period of 6–13 days (Figure 3).

A second analysis was conducted with additional incubation periods that were calculated by using the difference in time of onset between the first and second cases within 12 households. These additional data were added to the 16 data points from the first analysis. For the total 28 data points available for this second analysis, the mean incubation period was 8.3 days and median was 8 days. From the second analysis, the central 75% of case-patients had an incubation period of 5–12 days (Figure 3). For subsequent calculations in this investigation, we used the extremes in the 2 analyses as the incubation range (5–13 days, a range indicating the least and most number of days between exposure and onset of rash).

A third analysis of this data was performed by using the model described by Jezek et al. (*11*). In this analysis, which generates a model of serial intervals from the observed data, the transmission interval of the 16 well-described cases was 9.7 (95% CI 8.35–10.95) days; the interval for all 28 cases was 7.4 (95% CI 6.76–7.99) days.

### Transmission Chains

**Figure 4:**
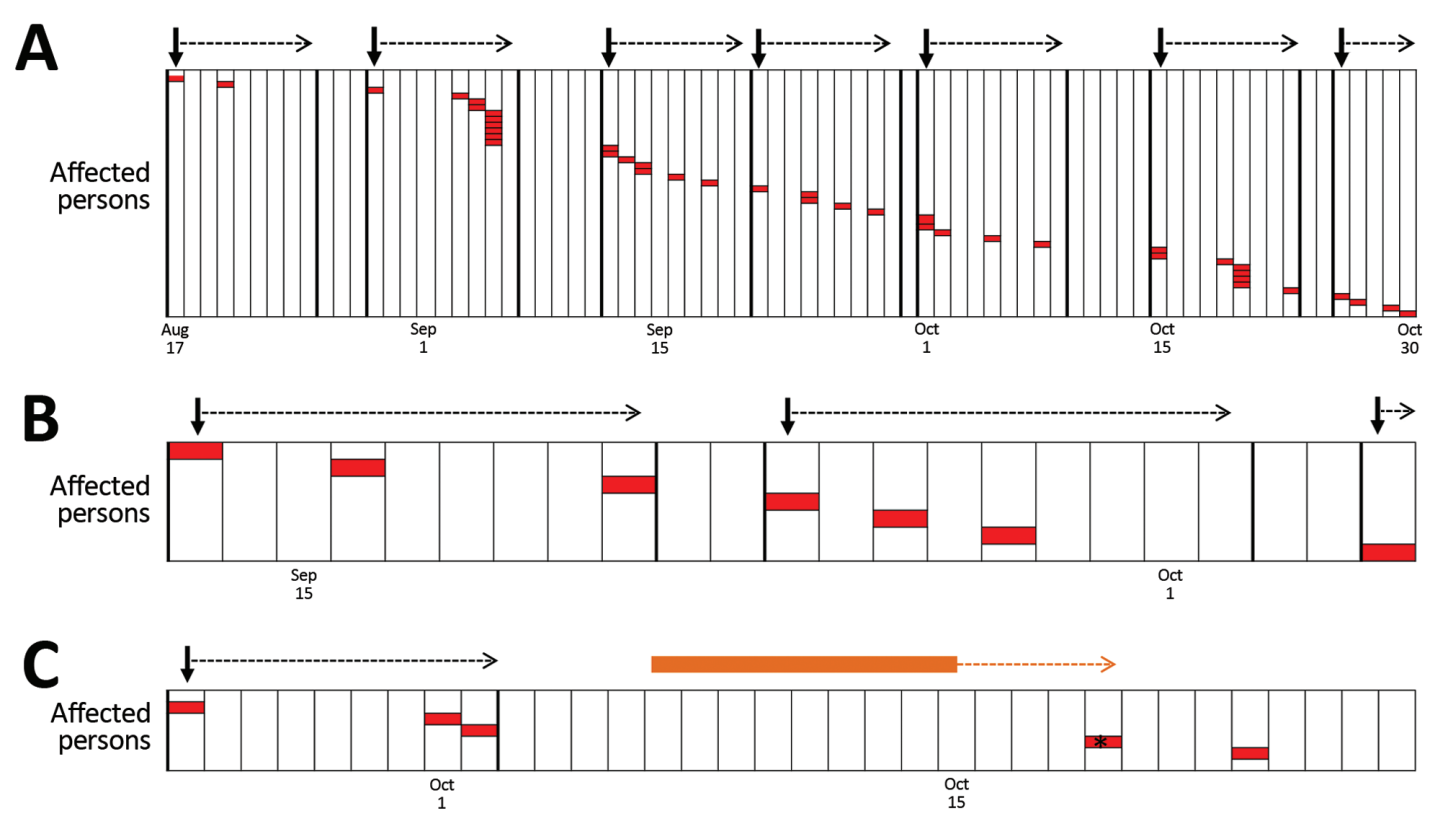
Reconstruction of monkeypox virus transmission events in the Democratic Republic of the Congo by using an estimated incubation period. Each column represents a calendar day. Red boxes represent a single case of monkeypox infection. A cluster is defined as a set of case-patients that could have resulted from a single exposure and are delimitated with dark vertical lines. Dark arrows indicate the first case within a cluster, and the dotted arrow indicates the time during which a potential single exposure could have produced symptoms in the first person in that group to the last (i.e., 5–13 days). A) Transmission events in the village of Bokungu in the Democratic Republic of the Congo. B) A household with evidence of 3 known transmission events. C) A household with evidence of 1 known and 1 unknown transmission event. The orange bar represents the days when the case-patient, represented by an asterisk (*), would be expected to have been exposed.

Using the range of 5–13 days as the incubation period, we reconstructed transmission events within families and villages. When the longest incubation period (13 days) was used, 9 of 16 households showed >1 transmission event. When the shortest time of incubation was used (5 days), an additional 4 households showed patterns consistent with transmission within the household (Figure 4, panel A). Two households had cases separated by a considerable period, suggesting the occurrence of either an unknown transmission event within the household or an exposure outside of the household (Figure 4, panel B). When community-wide transmission was considered within the health zone, longer transmission chains were observed, with the longest being in the village of Bokungu, where >7 suspected transmission events resulted in 42 apparent cases (Figure 4, panel C). Tests for spatial autocorrelation showed that case households for were more spatially clustered (z-scores >2.0) than would be expected randomly at all distances of 50–1,000 meters.

## Discussion

Human MPXV infection is endemic to DRC, with cases occurring throughout the Congo Basin. Many of these cases occur in isolation or in small clusters, but large outbreaks occasionally occur that involve many persons over a large geographic area. During 2013, a total of 104 cases of human monkeypox illness were reported in the Bokungu Health Zone. In contrast, only 17 cases were reported in 2011 and 13 in 2012. The surveillance system did not change substantively during this period; consequently, the rate in 2013 represents an increase of >600%, compared with rates for previous years. Our investigation focused on cases that occurred during the height of the 2013 outbreak.

Within the investigation period, 57% of affected persons were male, and median age was 10 years; 18% were <5 years of age. According to the United Nations World Population Prospects (http://esa.un.org/unpd/wpp/), 17.8% of the population in DRC is <5 years of age. Consequently, the age demographics of patients during the monkeypox outbreak correspond to those of the general population, suggesting that young children are not more prone to MPXV infection than others. A parallel study performed during this outbreak found no association between monkeypox illness and hunting or consumption of specific animals (*12*).

Previous publications have reported attack rates of 3%–11% (*6*,*8*); our investigation found a median attack rate of 50%, and 1 family had 100% of persons affected. The previously published attack rates are considerably lower than those for other viruses with similar routes of transmission; for example, smallpox has attack rates of 35%–88% (*13**–**15*), and variola virus had an attack rate of 90% (*16*). The difference between the findings reported here and those reported previously may result from several different causes. First, the high attack rate reported here possibly results from changing individual- and population-level immunity caused by elimination of routine childhood smallpox vaccination (*17*). Earlier investigations may have found lower attack rates because more persons had vaccine-derived immunity; however, some persons in the outbreak that we investigated had MPXV infection and prior smallpox vaccination, which suggest possible waning immunity over time, a factor that should be considered in future investigations. Second, a viral strain different from that found in previous investigations could have circulated in this outbreak and resulted in the high attack rate. However, we have no evidence for accepting or rejecting this possibility. Third, the high attack rate possibly reflects a high rate of case-patient identification in this investigation. We found that many persons are often affected in a household but that only 1 household member usually seeks medical attention, causing only 1 case to be recorded or investigated for surveillance. Case reporting on the basis of persons seeking healthcare may have caused the surveillance system to underestimate of the number of human monkeypox cases. Because we used in-home interviews, many previously unidentified cases were uncovered, enabling the calculation of a more accurate attack rate.

Previous investigations have shown a limited transmission capacity of MPXV within the human population. The highest number of suspected serial transmission events previously recorded is 6 (*4**,**9*). The ability to identify transmission events is limited by our lack of knowledge of the dynamics of infection. Often, whether 2 persons were co-infected by the same person and have different incubation periods or whether the persons were sequentially infected is difficult to determine. Understanding the incubation period of MPXV is vital for creating accurate transmission chains and determining if multiple introductions (human or zoonotic) occurred.

Sixteen patients with well-defined incubation periods were identified in our investigation and in the literature. Although these defined incubation periods are the best information available, they are also limited in number. Consequently, we also included apparent incubation periods within households. The time between onset of rash in 1 case and onset of rash in a subsequent case within a household provide an approximate incubation period. The longer that MPXV is present in a household, the more difficult identifying a clear infection chain is; therefore, only the transmission between the first and second case was used for the analysis. However, these 2 persons could have been infected by an outside source instead of by human-to-human transmission. The 3 analyses that we developed and presented here yielded similar results; 75% of the incubation periods were 5–13 days. Analysis of the same data by using the model proposed by Jezek et al. (*11*) yielded transmission intervals that matched data from our mathematical distribution model when we analyzed the 16 well-described cases. When all 28 cases were analyzed, the Jezek model produced an interval 1 day shorter than that for the mathematical distribution. The difference between these numbers likely results from the weighting that is included in the Jezek model. Further work is needed to evaluate which model best fits the biology of MPXV.

Our investigation suggests a shorter incubation period for MPXV than that observed in many animal models (*7**,**18**,**19*). Differences in organism and exposure may account for this difference. Experimental animals are often exposed to a virus for a brief time, and the interval between that exposure and development of symptoms is recorded as the incubation period. In contrast, the incubation period in this study was defined as the time between onset of rash for the first person infected and onset of rash in the second. Although high levels of viral shedding begin with the onset of rash, virus may be transmitted before the onset of rash. Research in prairie dogs has shown that oral shedding of virus begins before the development of dermal rash (*5*); this finding indicates that spread of MPXV is possible before the appearance of external skin lesions. Therefore, the time from first exposure to development of symptoms calculated in laboratory settings may be longer than the time calculated in this analysis.

Little information is available regarding the incubation period of MPXV in humans. A monkeypox outbreak in the United States revealed that the incubation period varied (range 12–14 days), and this period was dependent on the route and nature of exposure (*7*). All US cases resulted from exposure to infected pets. In contrast, the infections described in this article are likely caused by exposure to wild animals or an infected human. The type of exposure and route of virus transmission may result in incubation periods during the US outbreak that differ substantially from those observed in the outbreak that was the focus of our investigation. In addition, previous outbreaks were caused by viruses from a different genetic clade than that which caused the outbreak reported here. Transmission times may differ because of the specific virus involved.

Altogether, the Bokungu Health Zone had 42 cases in >7 infection clusters (i.e., a group of cases that could have resulted from a single infectious exposure). These clusters could have been created in 3 different ways. First, the clusters could be linked sequentially, whereby the infection could be externally introduced into a cluster and then passed by 1 person from that group to cluster 2 and so on. Second, transmission may not have occurred in a clear linear fashion, but persons may have had multiple human exposures. Third, MPXV could have been reintroduced into the community from an external source (zoonotic or human) during the course of the outbreak. Although we cannot determine which of these possibilities is most likely, we favor the second model because community interactions would make a strictly linear pattern of spread unlikely to occur. Further, the limited number of cases in the population as a whole makes it less likely that an external source was causing frequent reintroductions. We can conclude that >6 transmissions or introductions occurred in this health zone after the initial infection.

This report has limitations that should be considered. First, MPXV infection was laboratory confirmed in 48% of the cases by using PCR; the remaining cases were identified by patients’ symptoms. Laboratory confirmation was not possible for many cases because patients were interviewed after symptoms had resolved. Local resources for performing specimen collection were unavailable during all phases of the outbreak, so confirmation of MPXV infection for many cases was not possible. The lack of specimen collection has been noted as a limitation of the current surveillance program, and we are actively addressing this issue. Second, modeling of the incubation period was limited by the inability of most patients to identify a specific source of infection or a date of exposure. We assumed a minimum incubation period of 3 days when we created the incubation period model. This assumption was necessary to prevent bias toward very short incubation periods and is appropriate because of the longer incubation periods observed in animal models (*18**,**20*). Third, calculations were performed in our investigation with the assumption that transmission occurs once a person is symptomatic. Because data regarding transmission of MPXV are limited, this assumption was necessary; however, this assumption should be considered for evaluating incubation times and transmission.

This analysis provides insight into the dynamics of MPXV infection. We observed an average household attack rate of 50%, a much higher rate than reported in previous studies. Measures to decrease this attack rate should be implemented, including family-based education related to hygiene and isolation of patients. The transmission patterns observed in this outbreak also suggest transmission at the community level; therefore, community-wide education should begin as soon as the first monkeypox case is identified in an area. The calculated incubation period of 5–13 days further refines our understanding of the longest period of MPXV transmission risk after exposure in a natural setting. Knowledge of transmission risk is helpful for considering the appropriate monitoring period for exposed persons. This investigation and future work will improve our understanding of MPXV infection and our ability to limit its spread.

## References

[1] Damon IK. Status of human monkeypox: clinical disease, epidemiology and research. Vaccine. 2011;29(Suppl 4):D54–9. 10.1016/j.vaccine.2011.04.014 22185831

[2] Jezek Z, Fenner F. Human monkeypox. Monogr Virol. 1988;17:111–24.. 10.1159/000416463

[3] Jezek Z, Szczeniowski M, Paluku KM, Mutombo M. Human monkeypox: clinical features of 282 patients. J Infect Dis. 1987;156:293–8. 10.1093/infdis/156.2.293 3036967

[4] Formenty P, Muntasir MO, Damon I, Chowdhary V, Opoka ML, Monimart C, Human monkeypox outbreak caused by novel virus belonging to Congo Basin clade, Sudan, 2005. Emerg Infect Dis. 2010;16:1539–45. 10.3201/eid1610.100713 20875278 PMC3294404

[5] Hutson CL, Carroll DS, Gallardo-Romero N, Weiss S, Clemmons C, Hughes CM, Monkeypox disease transmission in an experimental setting: prairie dog animal model. PLoS One. 2011;6:e28295. 10.1371/journal.pone.0028295 22164263 PMC3229555

[6] Jezek Z, Grab B, Szczeniowski MV, Paluku KM, Mutombo M. Human monkeypox: secondary attack rates. Bull World Health Organ. 1988;66:465–70. 2844429 PMC2491159

[7] Reynolds MG, Yorita KL, Kuehnert MJ, Davidson WB, Huhn GD, Holman RC, Clinical manifestations of human monkeypox influenced by route of infection. J Infect Dis. 2006;194:773–80. 10.1086/505880 16941343

[8] Fine PE, Jezek Z, Grab B, Dixon H. The transmission potential of monkeypox virus in human populations. Int J Epidemiol. 1988;17:643–50. 10.1093/ije/17.3.643 2850277

[9] Learned LA, Reynolds MG, Wassa DW, Li Y, Olson VA, Karem K, Extended interhuman transmission of monkeypox in a hospital community in the Republic of the Congo, 2003. Am J Trop Med Hyg. 2005;73:428–34. 16103616

[10] Kulesh DA, Loveless BM, Norwood D, Garrison J, Whitehouse CA, Hartmann C, Monkeypox virus detection in rodents using real-time 3′-minor groove binder TaqMan assays on the Roche LightCycler. Lab Invest. 2004;84:1200–8. 10.1038/labinvest.3700143 15208646 PMC9827366

[11] Jezek Z, Arita I, Mutombo M, Dunn C, Nakano JH, Szczeniowski M. Four generations of probable person-to-person transmission of human monkeypox. Am J Epidemiol. 1986;123:1004–12. 3010703 10.1093/oxfordjournals.aje.a114328

[12] Nolen LD, Osadebe L, Katomba J, Likofata J, Mukadi D, Monroe B, Introduction of monkeypox into a community and household: risk factors and zoonotic reservoirs in the Democratic Republic of the Congo. Am J Trop Med Hyg. 2015;93:410–5. 10.4269/ajtmh.15-0168 26013374 PMC4530773

[13] Thomas DB, McCormack WM, Arita I, Khan MM, Islam S, Mack TM. Endemic smallpox in rural East Pakistan. I. Methodology, clinical and epidemiologic characteristics of cases, and intervillage transmission. Am J Epidemiol. 1971;93:361–72. 5556397 10.1093/oxfordjournals.aje.a121269

[14] Rao AR, Jacob ES, Kamalakshi S, Appaswamy S, Bradbury. Epidemiological studies in smallpox. A study of intrafamilial transmission in a series of 254 infected families. Indian J Med Res. 1968;56:1826–54. 5732451

[15] Fenner F, Henderson DA, Arita I, Ježek Z, Ladnyi ID. Smallpox and its eradication. Geneva: World Health Organization; 1988.

[16] Shrivastava SR, Shrivastava PS, Ramasamy J. Epidemiological investigation of a case of chickenpox in a medical college in Kancheepuram, India. Germs. 2013;3:18–20. 10.11599/germs.2013.1032 24432282 PMC3882843

[17] Rimoin AW, Mulembakani PM, Johnston SC, Lloyd Smith JO, Kisalu NK, Kinkela TL, Major increase in human monkeypox incidence 30 years after smallpox vaccination campaigns cease in the Democratic Republic of Congo. Proc Natl Acad Sci U S A. 2010;107:16262–7. 10.1073/pnas.1005769107 20805472 PMC2941342

[18] Hutson CL, Carroll DS, Self J, Weiss S, Hughes CM, Braden Z, Dosage comparison of Congo Basin and West African strains of monkeypox virus using a prairie dog animal model of systemic orthopoxvirus disease. Virology. 2010;402:72–82. 10.1016/j.virol.2010.03.012 20374968 PMC9533845

[19] Goff AJ, Chapman J, Foster C, Wlazlowski C, Shamblin J, Lin K, A novel respiratory model of infection with monkeypox virus in cynomolgus macaques. J Virol. 2011;85:4898–909. 10.1128/JVI.02525-10 21389129 PMC3126178

[20] Nagata N, Saijo M, Kataoka M, Ami Y, Suzaki Y, Sato Y, Pathogenesis of fulminant monkeypox with bacterial sepsis after experimental infection with West African monkeypox virus in a cynomolgus monkey. Int J Clin Exp Pathol. 2014;7:4359–70. 25120821 PMC4129056

