# Correction: Vol. 22, No. 6

**DOI:** 10.3201/eid2210.C22210

**Published:** 2016-10

**Keywords:** errata, erratum, correction

The increase in monkeypox cases in the Bokungu Health Zone of the Democratic Republic of the Congo during the second half of 2013 should have been listed as >600% in the abstract of the article in Extended Human-to-Human Transmission during a Monkeypox Outbreak in the Democratic Republic of the Congo (L.D. Nolen al.). The article has been corrected online (http://wwwnc.cdc.gov/eid/article/22/6/15-0579_article).

